# Deciphering microbial diversity associated with *Fusarium* wilt-diseased and disease-free banana rhizosphere soil

**DOI:** 10.1186/s12866-019-1531-6

**Published:** 2019-07-12

**Authors:** Dengbo Zhou, Tao Jing, Yufeng Chen, Fei Wang, Dengfeng Qi, Renjun Feng, Jianghui Xie, Huaping Li

**Affiliations:** 1 Institute of Tropical Bioscience and Biotechnology, China Academy of Tropical Agricultural Sciences, Haikou, Hainan China; 2 Haikou Experimental Station, China Academy of Tropical Agricultural Sciences, Haikou, Hainan China; 3 Guangdong Province Key Laboratory of Microbial Signals and Disease Control, College of Agriculture, South China Agricultural University, Guangzhou, Guangdong China

**Keywords:** Banana *Fusarium* wilt, Bacterial and fungal communities, Pathogen abundance, Environmental variables

## Abstract

**Background:**

*Fusarium* wilt of banana (*Musa* spp.) caused by the fungal pathogen *Fusarium oxysporum* f. sp. *cubense* (*Foc*) is a typical soilborne disease, that severely devastates the banana industry worldwide, and soil microbial diversity is closely related to the spread of *Fusarium* wilt. To understand the relationship between microbial species and *Fusarium* wilt, it is important to understand the microbial diversity of the *Fusarium* wilt-diseased and disease-free soils from banana fields.

**Results:**

Based on sequencing analysis of the bacterial 16S *rRNA* genes and fungal internal transcribed spacer (ITS) sequences, *Foc* abundance, fungal or bacterial richness and diversity were higher in the diseased soils than in the disease-free soils. Although *Ascomycota* and *Zygomycota* were the most abundant fungi phyla in all soil samples, *Ascomycota* abundance was significantly reduced in the disease-free soils. *Mortierella* (36.64%) was predominant in the disease-free soils. Regarding bacterial phyla, *Proteobacteria*, *Acidobacteria*, *Chloroflexi*, *Firmicutes*, *Actinobacteria*, *Gemmatimonadetes*, *Bacteroidetes*, *Nitrospirae*, *Verrucomicrobia* and P*lanctomycetes* were dominant phyla in all soil samples. In particular, *Firmicutes* contributed 16.20% of the total abundance of disease-free soils. At the bacterial genus level, *Bacillus*, *Lactococcus* and *Pseudomonas* were abundant in disease-free soils with abundances of 8.20, 5.81 and 2.71%, respectively; lower abundances, of 4.12, 2.35 and 1.36%, respectively, were found in diseased soils. The distribution characteristics of fungal and bacterial genera may contribute to the abundance decrease of *Foc* in the disease-free soils.

**Conclusion:**

Unique distributions of bacteria and fungi were observed in the diseased and disease-free soil samples from banana fields. These specific genera are useful for constructing a healthy microbial community structure of soil.

## Background

Microorganisms play an important role in the formation and maintenance of healthy soil, improve soil conditions through many enzymatic activities that regulate the biogeochemical cycle, reorganization and mineralization of organic matter [1–6]. The microbiota present in soil are affected by climate, cultivation methods, soil nutrients, pathogens and soil management practices [7–10]. Rhizosphere microorganisms comprise a dynamic community with a complex interaction with plants [11]. Some intrinsic microbial communities or specific sub-populations have potential functions to suppress soilborne diseases caused by fungi, oomycetes, bacteria and nematodes [5, 6, 11]. Hence, understanding the microbial composition of the plant rhizosphere is key to controlling the spread of soilborne disease.

Banana (*Musa* spp.) is an important cash and food crop in the tropics and subtropics and is one of the world’s top 10 staple foods [12, 13]. Most of cultivated bananas multiplied by the vegetative propagation are prone to infection by various pests and diseases, particularly *Fusarium* wilt of banana. The disease is caused by the soilborne fungus *Fusarium oxysporum* f. sp. *cubense* (*Foc*), which is one of the major constraints to banana production worldwide [14]. *Foc* infects the xylem through the roots, and causes extensive necrosis leading to of plant deaths [14]. Currently, management of the *Fusarium* wilt disease mainly involves rotation, selection of resistant varieties, and chemical or biological methods. Nonetheless, there is no an effective control for *Fusarium* wilt of banana, as evidenced by the continuous spread among continents, countries and regions [15, 16]. In general, biocontrol is considered as a safe, environmentally friendly and cost-effective method for disease control [5, 8, 16].

Multiple pathogen-plant systems have been widely used to suppress different innate diseases in the agricultural soil [6, 17–19]. Once attacked by root pathogens, plants can exploit microbial consortia from soil for protection against pathogen infection. Comparative microbiome analysis indicated that the relative abundance of bacterial and fungal diversity was significantly higher in the suppressive soil than that in the conducive soil [6]. High richness and diversity indices of bacterial communities were also detected in the soil naturally suppressive to *Fusarium* wilt of banana [18]. In addition, known plant-beneficial rhizobacteria such as *Azospirillum*, *Gluconacetobacter*, *Burkholderia*, *Comamonas* and *Sphingomonadaceae* were more prevalent in soil suppressive to tobacco black root rot disease [19]. Furthermore, application of *Paenibacillus polymyxa* NSY50 reduced the abundance of *Fusarium* and increased the population of beneficial microbes, including *Bacillus, Actinobacteria, Streptomyces, Actinospica, Catenulispora* and *Pseudomonas* genera [20]. These beneficial microorganisms inhibit pathogen infection through metabolites such as pyoverdins and iron-chelating siderophores [21]. Hence, novel bacterial and fungal taxa can serve as indicators of disease suppression in soil-quality assessments. A previous study revealed that microbial species composition and abundance, which are also significantly influenced by the chemical properties of soil, are vital for favorable microecology [22]. However, how soil microbial species are affected by mutual constraints among disease occurrence, pathogenic abundance, soil chemical properties and beneficial microorganisms are still an open question.

In the present study, we performed a comparative microbiome analysis of the *Fusarium* wilt-diseased and *Fusarium* wilt-suppressive (disease-free) rhizosphere soils. Based on 454-pyrosequencing of the fungal internal transcribed spacer (ITS) region and the bacterial 16S *rRNA* gene, changes in dominant bacterial and fungal species were investigated in both types of soils. Additionally, correlations between soil chemical properties and microbial distributions were analyzed. Our results offer new insight into identifying fungal and bacterial genera associated with *Fusarium* wilt disease-free soils and suggest the microbial genera and mechanisms involved in *Fusarium* wilt suppressiveness.

## Results

### Quantitative analysis of *Foc* colonization

**Fig. 1:**
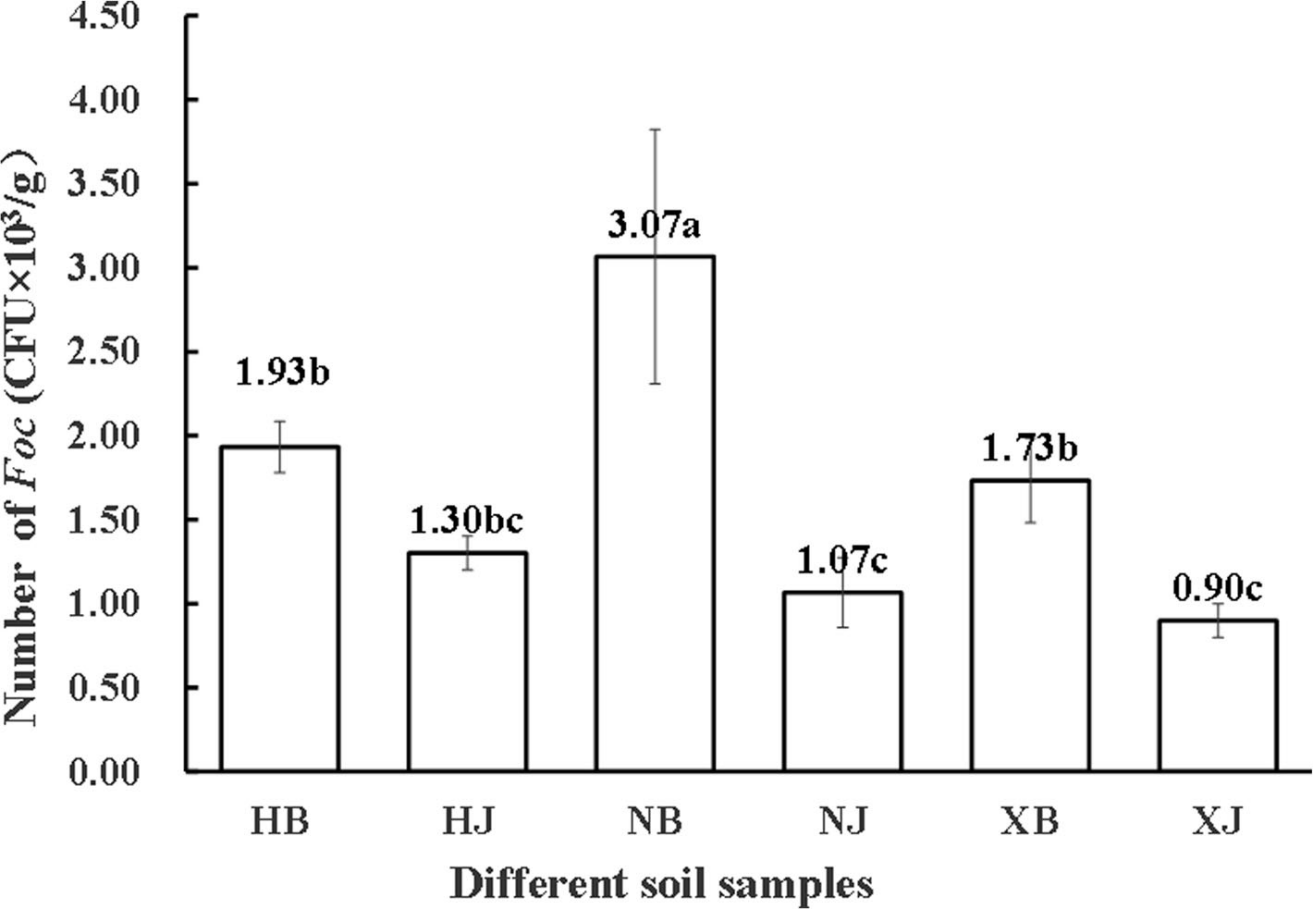
Number of the pathogen *Fusarium oxysporum* f. sp. *cubense* in different soil samples. HB, NB and XB represent the diseased soil samples collected from Huangtong, Nanbao and Xinying farms, respectively. HJ, NJ and XJ represent the disease-free soil samples collected from Huangtong, Nanbao and Xinying farms, respectively. The data in each column is the average value of three replicates (*n* = 10). The letter on each bar represents a significant difference at the 5% level

*Fusarium* wilt-diseased soil samples (XB + HB + NB) and disease-free soil samples (XJ + HJ + NJ) were collected from three farms. Abundance of *Foc* was analyzed in each soil sample. Our results showed that population of *Foc* ranged from1.73 × 10^3^ CFU/g to 3.07 × 10^3^ CFU/g in *Fusarium* wilt-diseased soils and from 0.90 × 10^3^ CFU/g to 1.30 × 10^3^ CFU/g in the diseased-free soils. The largest population of *Foc* (3.07 × 10^3^ CFU/g) was detected in the NB sample and the smallest (0.90 × 10^3^ CFU/g) in the XJ sample. Quantitative analysis of *Foc* colonization showed more than 1700 CFU/g in the diseased soil samples from the three different farms, but less than 1300 CFU/g of *Foc* was detected in the disease-free soils. On the same farm, the *Foc* population in the diseased soil samples (HB, NB and XB) were higher (*p* < 0.05) than those in the disease-free soil samples (HJ, NJ and XJ) (Fig. 1).

### Microbiome data acquisition and analysis

After quality evaluation of raw sequence libraries, a pyrosequencing-based analysis was performed to detect the V3-V4 regions of the bacterial 16S *rRNA* genes and the fungal ITS sequences. In total, we obtained 540,711 high-quality bacterial sequences and 542,218 fungal sequences from 18 soil samples. The high-quality reads ranged from 20,119 to 38,942 for bacterial datasets and from 21,437 to 37,800 for fungal datasets. The length distribution of trimmed sequences ranged from 304 bp to 509 bp for bacterial datasets and from 201 bp to 356 bp for fungal datasets. After homogenization, a total of 444,817 high-quality bacterial 16S *rRNA* sequences were clustered into 3368 distinct bacterial Operational Taxonomic Units (OTUs). Regarding fungal communities, 1517 distinct OTUs were observed for 380,736 high-quality fungal ITS sequences. Rarefaction curves were drawn for the fungal and bacterial datasets according to OTUs. These data had been submitted to NCBI under accession number SRP132524.

### Richness and diversity of bacterial and fungal communities

**Fig. 2:**
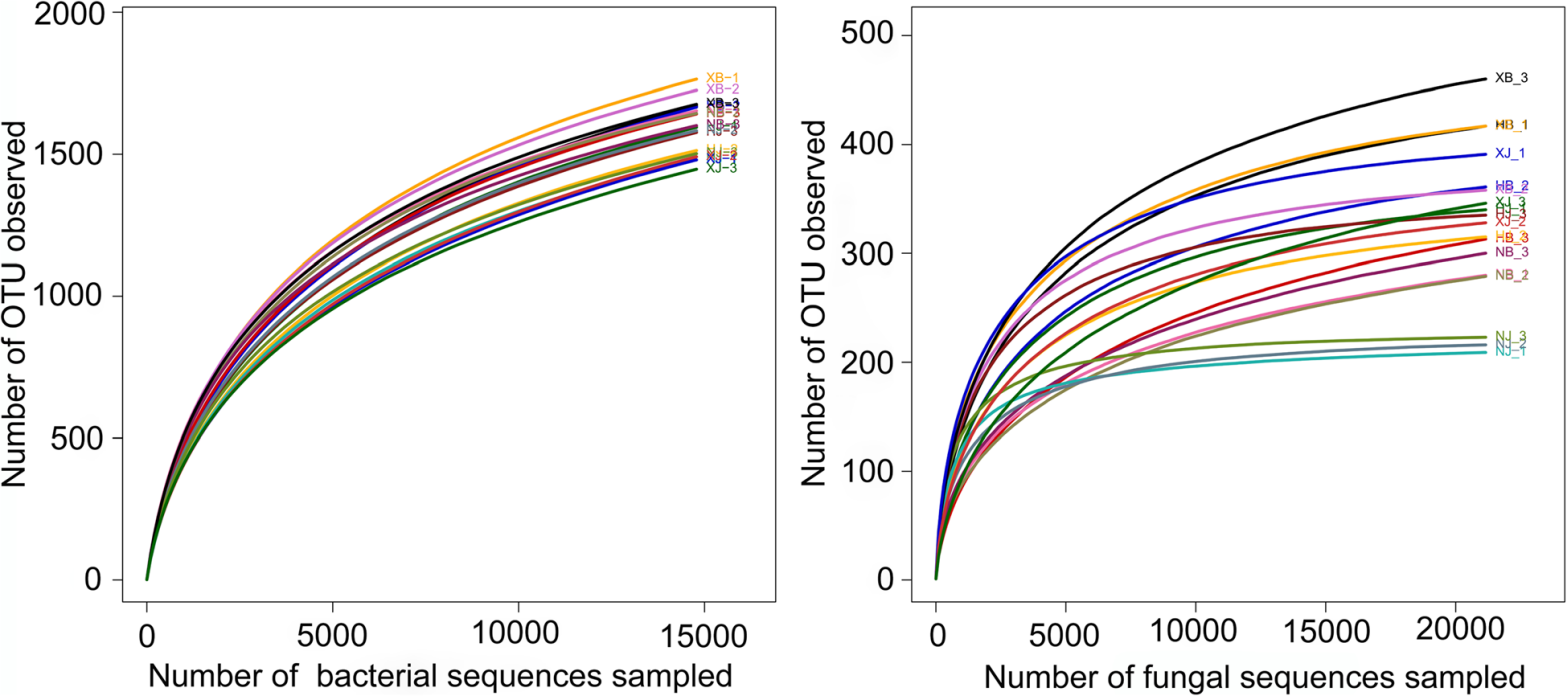
Rarefaction analysis of the diseased and disease-free soil samples at a 3% dissimilarity level. HB, NB, XB, HJ, NJ and XJ were described in Fig. 1. Each sample had three replicates (*n* = 10)

Pooled sequences from three replicates for each sample were compared at 3% dissimilarity. A similar tendency of OTUs change was found for the diseased and disease-free soil samples from the same farm (Fig. 2). The diseased soil samples (NB and HB) showed higher OUTs than those in the disease-free soil samples (NJ and HJ). Similarly, higher indices of the fungal ACE and Chao1 were observed in the diseased soil samples (Table 1). In contrast, no obvious difference (*p* < 0.05) in species diversity according to Shannon and Simpson indices was observed between the soil types, whereas a lower bacterial Simpson index was demonstrated in the diseased soil samples (Table 2). The disease-free soil samples owned higher indices (*p* < 0.05) of ACE, Chao1 and Shannon. For these bacterial sequences, the diseased soil samples exhibited higher OTUs than in the disease-free soil samples. For the fungal ITS sequences, higher OUT numbers were observed in the diseased soil samples (NB and HB) than those in the disease-free soil samples (NJ and HJ). However, opposite OUT number results were observed for XJ and XB samples.

**Table 1:** Evaluation of fungal diversity for six banana soil samples from the three banana farms

Soil samples	ACE	Shannon	Chao1	Simpson
HB	417.33 (37.072)^ab^	3.07 (0.47)^b^	422.01(52.47)^ab^	0.132 (0.0569)^ab^
HJ	342.33 (4.73)^c^	3.08 (0.35)^b^	360.15 (18.02)^b^	0.1982 (0.0759)^a^
NB	353.00 (14.80)^bc^	3.09 (0.08)^b^	399.46 (8.01)^ab^	0.0962 (0.0183)^ab^
NJ	221.33 (5.03)^d^	3.45 (0.49)^ab^	221.92 (4.74)^c^	0.0746 (0.0100)^ab^
XB	450.33 (75.66)^a^	4.01 (0.03)^a^	450.56 (75.51)^a^	0.042 (0.0022)^b^
XJ	395.33 (33.65)^abc^	3.06 (0.77)^b^	395.66 (37.46)^ab^	0.1726 (0.1361)^ab^

Note: HB, NB, HJ, NJ, XJ and XB are described in Fig. 1. Values are means followed by a standard error in the brackets. Each experiment was repeated for three times. The letter in the column represents a significant difference (Tukey HSD test, *p* < 0.05)

**Table 2:** Evaluation of bacterial diversity for six banana soil samples from the three banana farms

Soil samples	ACE	Shannon	Chao1	Simpson
HB	2126.7 (53.26)^a^	6.21 (0.038)^b^	2375.52 (39.34)^a^	0.0062 (0.0006)^bc^
HJ	1996 (55.65)^c^	6.02 (0.098)^c^	2125.27 (57.97)^bc^	0.0083 (0.0012)^ab^
NB	2007.7 (27.72)^b^	6.46 (0.079)^a^	2175.09 (27.41)^bc^	0.0032 (0.0006)^c^
NJ	1941.3 (45.24)^c^	6 (0.178)^c^	2050.83 (104.06)^c^	0.0096 (0.0039)^a^
XB	2148.3 (60.07)^a^	6.49 (0.01)^a^	2257.17 (119.89)^ab^	0.0036 (0.0001)^c^
XJ	1986.3 (38.94)^c^	5.9 (0.026)^c^	2105.34 (47.12)^c^	0.0085 (0.0000)^ab^

Note: HB, NB, HJ, NJ, XJ and XB are described in Fig. 1. Values are means followed by a standard error in the brackets. Each experiment was repeated for three times. The letter in the column represents a significant differences (Tukey HSD test, *p* < 0.05)

### Distributions of bacterial and fungal communities

**Fig. 3:**
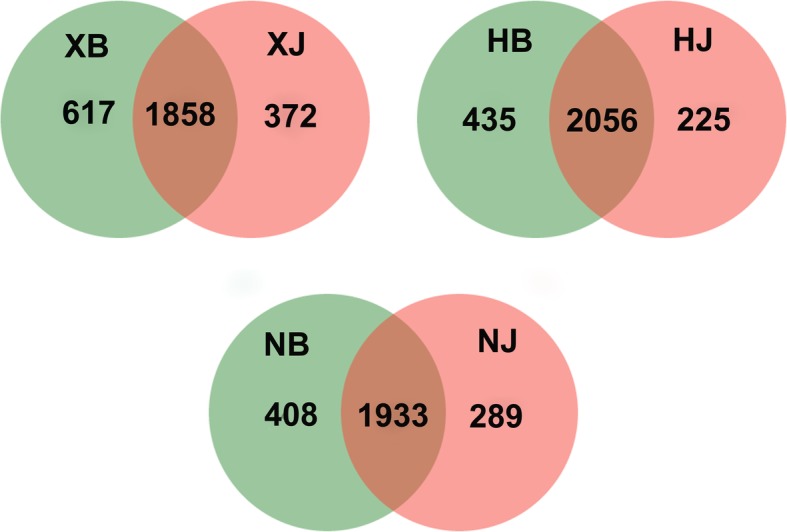
Venn diagram of bacterial disease and disease-free soil samples from the three different farms. Diseased soil samples (XB + HB + NB) and disease-free soil samples (XJ + HJ + NJ) were collected from different farms. The shared and unique bacteria OTUs were shown at a 0.03 dissimilarity distance after removing singletons

**Fig. 4:**
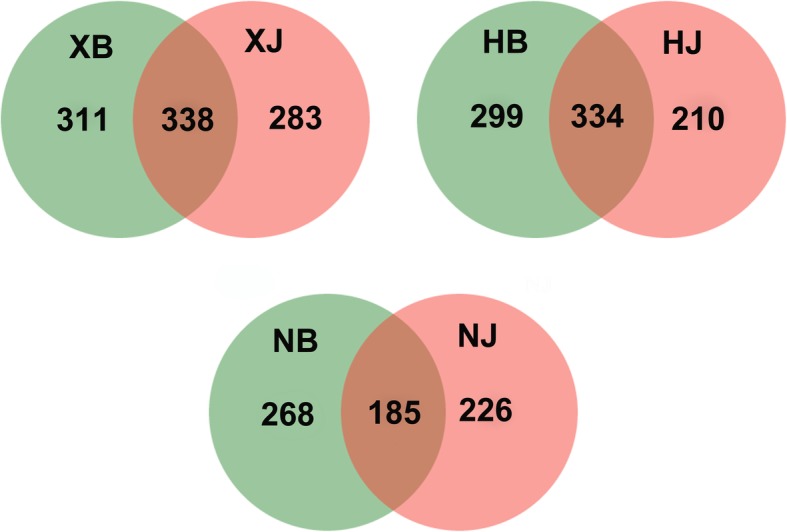
Venn diagram for fungal disease and disease-free soil samples from the three different farms. Diseased soil samples (XB + HB + NB) and disease-free soil samples (XJ + HJ + NJ) were collected from different farms. The shared and unique fungus OTUs were shown at a 0.03 dissimilarity distance after removing singletons

**Fig. 5:**
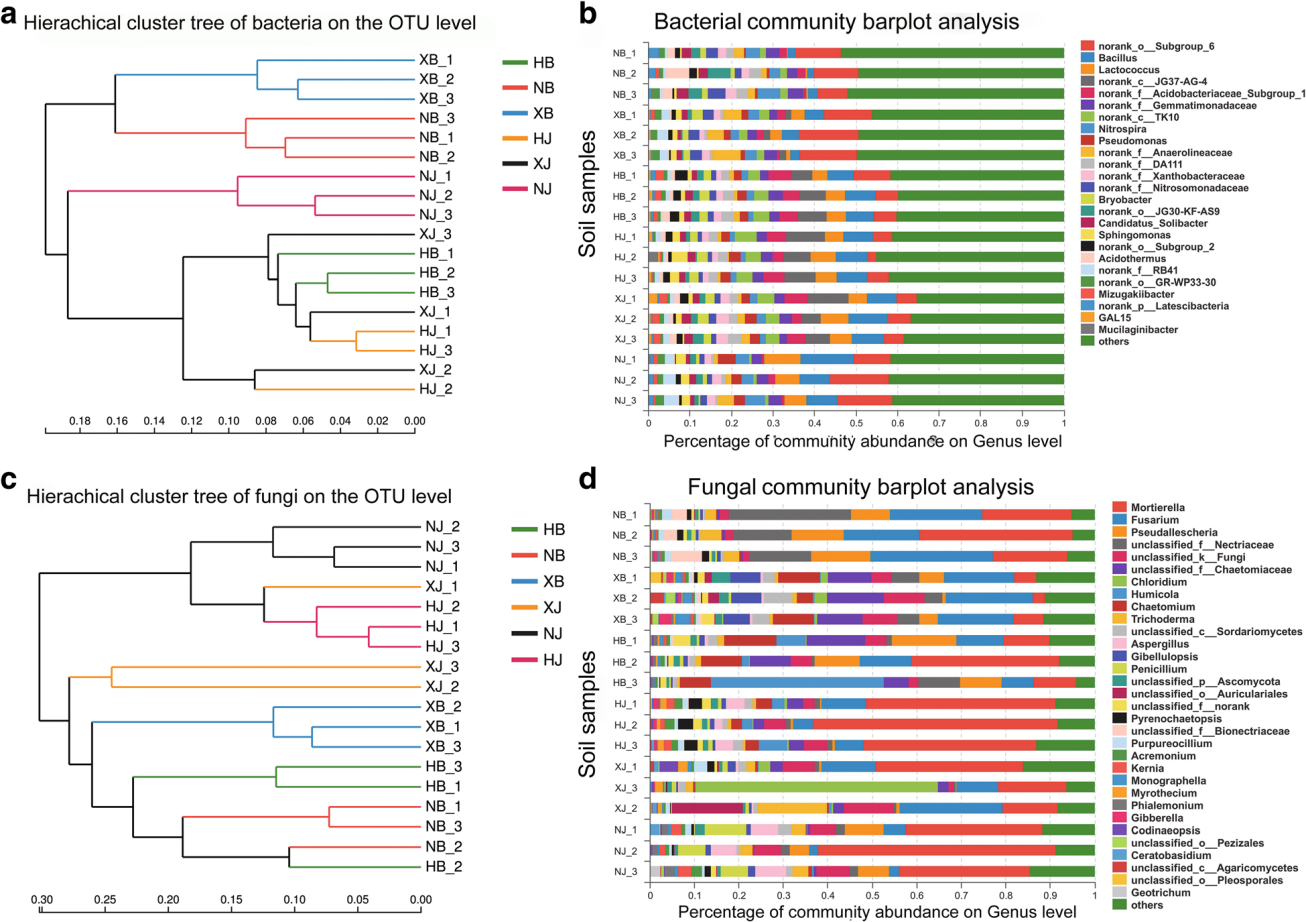
Construction of hierarchical cluster trees using a weighted UniFace algorithm for the diseased and the disease-free soil samples. Each sample has three replicates. **a** hierarchical cluster tree of bacteria; **b** abundance of bacterial community at the level of genus; **c** hierarchical cluster tree of fungi; **d** abundance of fungal community at the level of genus. “Others” indicate the abundance of less than 2% of genus

After removing singletons, 2475, 2491, 2341, 2281, 2222 and 2230 bacterial OTUs were obtained for XB, HB, NB, HJ, NJ and XJ, respectively. For fungal sequences, 633, 453, 649, 544, 411 and 621 OTUs were found in HB, NB, XB, HJ, NJ and XJ, respectively (Figs. 3, 4). In general, there were a greater number of bacterial and fungal OTUs in the diseased soil samples than in the disease-free soil samples from the same farm. Hierarchical cluster analysis revealed that the same farm presented the similar bacterial and fungal distributions (Fig. 5a and c), and bar plot analysis of the community structure further showed 25 bacterial and 32 fungal genera among the different farms (Fig. 5b and d). Although no obvious difference was observed in the number of genera between the diseased soils and the disease-free soils, the community distribution of unique genera was significantly different (*p* < 0.05).

### Bacterial and fungal community composition

**Fig. 6:**
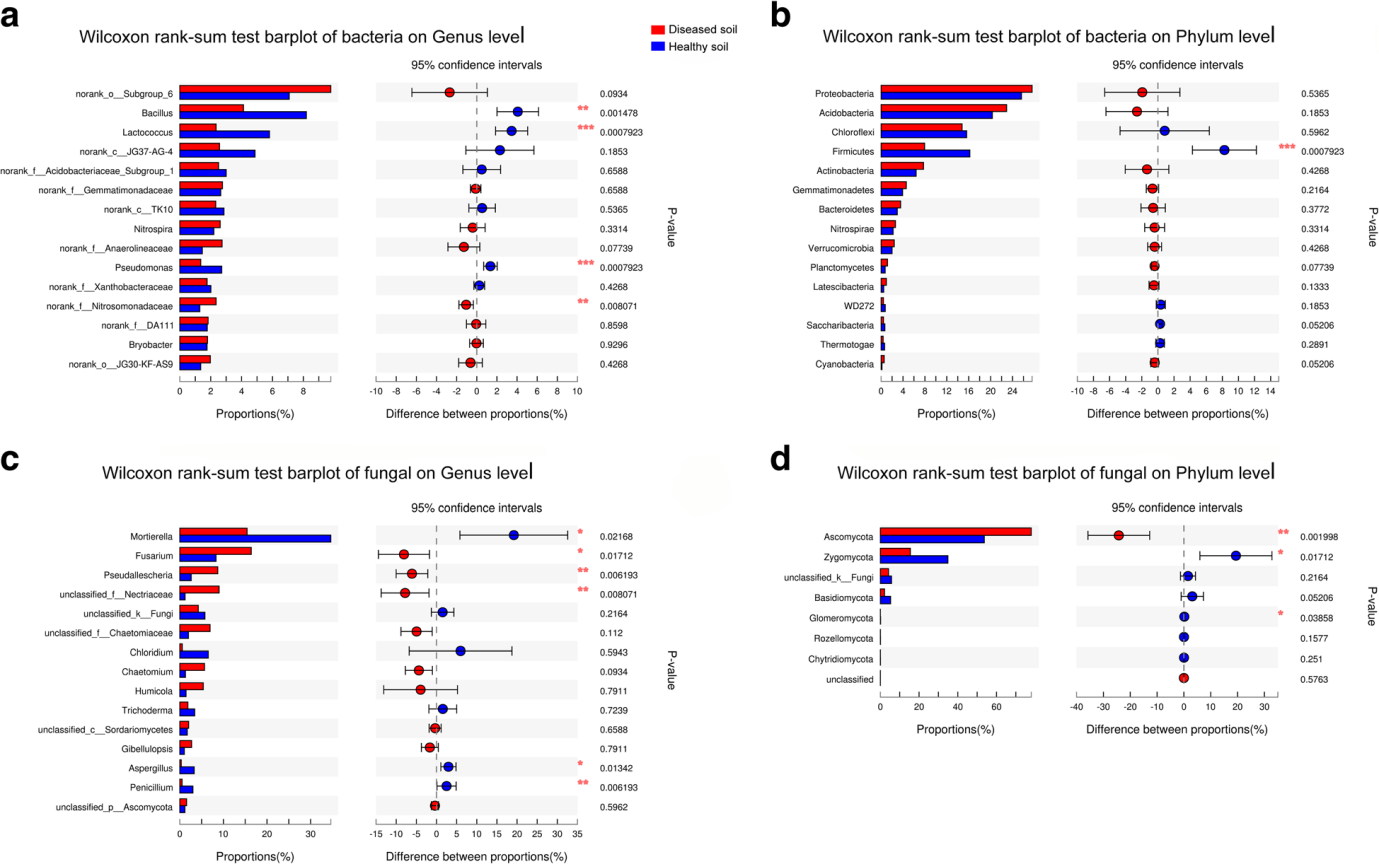
Analysis of abundance differences between the diseased soil samples and the disease-free soil samples at the bacterial genus. **a** richness differences of bacterial genus; **b** richness differences of bacterial phylum; **c** richness differences of fungal genus; **d** richness differences of fungal phylum. The *y*-axis represents the classification levels of species, and the *x*-axis represents the percentage values of species abundance in each sample. The blue and red columns represent the average results in the diseased and disease-free soil samples, respectively. Significant differences were showed according to the Wilcoxon rank-sum test (*: 0.01 < *P* < =0.05, **: 0.001 < *P* < =0.01, ***: *P* < =0.001)

The top 15 dominant bacterial genera and phyla were identified according to the total relative abundances of each genus and phylum in all samples (Fig. 6a, b). At the genus level, *Bacillus*, *Lactoccocus*, JG37-AG-4, *Acidobacteriaceae*, *Nitrospira*, *Anaerolineaceae*, *Pseudomonas*, *Xanthobacteraceae*, *Nitrosomonadaceae*, DA111, *Bryobacter* and JG30-KF-AS9 were dominant genera with over 1% of abundance in the diseased and disease-free soils. Four dominant genera (*Bacillus*, *Lactococcus*, *Pseudomonas*and, *Nitrosomonadaceae*) exhibited different proportions in both types of soil samples (Fig. 6a). At the phylum level, *Proteobacteria*, *Acidobacteria*, *Chloroflexi*, *Firmicutes*, *Actinobacteria*, *Gemmatimonadetes*, *Bacteroidetes*, *Nitrospirae*, *Verrucomicrobia* and *Planctomycetes* were identified as the dominant phyla. *Firmicutes* showed a proportional difference (*p* = 0.00079) between both types of soil samples. (Fig. 6b).

For the top 15 dominant fungal genera and phyla (Fig. 6c, d), *Mortierella*, *Fusarium*, *Pseudallescheria*, *Nectriaceae*, *Chloridium, Chaetomium*, *Humicola*, *Trichoderma*, *Ascomycota* were the dominant fungal genera in all soil samples (Fig. 6c), with relative abundance of more than 1%. Higher proportions of *Mortierella*, *Fusarium*, *Pseudallescheria*, *Nectriaceae*, *Aspergillus* and *Penicillium* were demonstrated. At the phylum level, *Ascomycota* and *Zygomycota* were considered to be dominant phyla and showed an obvious difference between the diseased soil samples and the disease-free soil samples (Fig. 6d).

### Comparison of fungal and bacterial community characteristics

For fungi, *Mortierella*, *Fusarium*, *Pseudallescheria* and *Nectriaceae* with over 0.1% of relative abundance were enriched in the diseased and disease-free soil samples. An obvious difference was observed in their relative abundance between both types of soils. In contrast, *Fusarium, Pseudallescheria* and *Nectriaceae* were enriched in the diseased soils with the abundances of 16.39, 8.71 and 9.00%, respectively, whereas only 8.30, 2.62 and 1.20% were detected in the disease-free soils, respectively. The most abundant genus *Mortierella* (34.64%) was detected in the disease-free soils, with only 15.44% abundance in the diseased soils. In addition, we found that the relative abundances of *Aspergillus* and *Penicillium* were 3.30 and 3.01%, respectively, in the disease-free soil samples, and 0.34 and 0.52%, respectively, in the diseased soil samples (Table 3).

**Table 3:** Comparison of the most abundant bacterial and fungal genera in both types of soil samples from the three banana farms

Genus	Diseased soil samples	Disease-free soil samples	*p*-value
Bacteria			
*Bacillus*	4.1220 (2.2550)	8.1950 (1.8620)	0.001478
*Lactococcus*	2.3460 (1.8090)	5.8090 (1.3470)	0.0007923
*Pseudomonas*	1.3580 (0.6663)	2.714 (0.6724)	0.0007923
*Nitrosomonadaceae*	2.3460 (0.9739)	1.291 (0.2796)	0.008071
*Sphingomonas*	0.9737 (0.3830)	1.9080 (0.9412)	0.02168
*Psychrobacter*	0.4367 (0.3985)	1.0670 (0.2313)	0.002626
*Acidobacteria*	0.6692 (0.1626)	0.4378 (0.1386)	0.01712
*Oceanobacillus*	0.2436 (0.202)	0.5771 (0.1673)	0.00268
*Brochothrix*	0.1873 (0.1751)	0.4345 (0.0855)	0.008039
*Parcubacteria*	0.3401 (0.3316)	0.0923 (0.1211)	0.03407
*Terrimonas*	0.1998 (0.0829)	0.09101 (0.0949)	0.02728
*Carnobacterium*	0.0738 (0.0652)	0.1904 (0.05604)	0.001998
*Flavisolibacter*	0.0571 (0.0387)	0.1259 (0.0753)	0.03407
*Streptococcus*	0.0446 (0.0327)	0.1247 (0.04781)	0.0004123
*Burkholderia*	0.0518 (0.0282)	0.1125 (0.06024)	0.01712
*Fungi*			
*Mortierella*	15.4400 (11.8100)	34.6400 (14.8200)	0.0217
*Fusarium*	16.3900 (6.0990)	8.3030 (6.5870)	0.0171
*Pseudallescheria*	8.7130 (4.4960)	2.6180 (3.2750)	0.0062
*Nectriaceae*	9.0030 (8.3860)	1.2040 (0.5967)	0.0081
*Aspergillus*	0.3379 (0.2652)	3.2980 (2.6480)	0.0134
*Penicillium*	0.5191 (0.2949)	3.0120 (3.349)	0.0062
*Ceratobasidium*	0.0687 (0.1635)	0.5849 (0.7355)	0.0080
*Cryptococcus*	0.1122 (0.0641)	0.3831 (0.1901)	0.0341
*Guehomyces*	0.0610 (0.0763)	0.3031 (0.3129)	0.0216

Note: The Wilcoxon rank-sum test method was used to compare differences of richness of the fungal and bacterial genus between the diseased soil samples and the disease-free soil samples. More than 0.1% abundances of classified genera were shown in the three farms. Values are means followed by a standard error in the brackets. The *p*-values indicate significant differences at the levels of 0.01, 0.05 and 0.001

Significant differences between the diseased and disease-free soils were found for 15 bacterial genera, with a relative abundance of more than 0.1%. Compared with the diseased soils, *Bacillus, Lactococcus*, *Pseudomonas*, *Sphingomonas*, *Psychrobacter*, *Oceanobacillus*, *Brochothrix*, *Carnobacterium*, *Flavisolibacter*, *Streptococcus* and *Burkholderia* were abundant in the disease-free soils. *Bacillus*, *Lactococcus* and *Pseudomonas* exhibited abundances of 8.20, 5.81 and 2.71% in the disease-free soils, respectively, and 4.12, 2.35 and 1.36% in the diseased soils. The abundance of *Streptococcus* in the diseased and disease-free soils was also significantly different (Table 3).

### Effects of soil environmental variables on phylum abundance

To understand the effects of soil environmental variables on the phylum abundances of bacteria and fungi, we measured total organic carbon (TOC), total organic nitrogen (TON), pH, available phosphorus (AP) and available potassium (AK) in the different soil samples. For a given farm, the disease-free soils had higher pH and AP (*p* < 0.05), while concentrations of TON, TOC and AK in the diseased soils were higher (*p* < 0.05) (Table 4).

**Table 4:** Measurement of chemical properties in six different soil samples from the three banana farms

Sample	pH	TON(mg·kg^−1^)	TOC (mg·kg^−1^)	AP (mg·kg^−1^)	AK(mg·kg^−1^)
HB	5.40 ± 0.06d	1480.76 ± 28.83b	39.45 ± 1.99b	18.18 ± 2.90e	2029.97 ± 19.56d
HJ	5.79 ± 0.06c	1324.64 ± 23.18d	35.41 ± 2.71c	81.64 ± 1.01b	1602.63 ± 29.16c
NB	5.22 ± 0.07e	1831.68 ± 36.95a	43.15 ± 1.45a	52.11 ± 7.74d	2490.59 ± 74.06b
NJ	6.07 ± 0.11a	931.21 ± 25.57f	26.57 ± 1.09d	114.71 ± 5.90a	2143.54 ± 51.53e
XB	5.40 ± 0.05d	1413.33 ± 38.60c	42.05 ± 0.50ab	65.28 ± 5.04c	2847.69 ± 72.78a
XJ	5.95 ± 0.05b	1136.72 ± 2.76e	34.48 ± 0.93c	113.52 ± 5.44a	1628.67 ± 59.74e

Note: HB, NB, HJ, NJ, XJ and XB were described in Fig. 1. Values represent means followed by a standard error. Each experiment was repeated for three times. The letter in the column represents a significant difference (Tukey HSD test, *p* < 0.05)

**Fig. 7:**
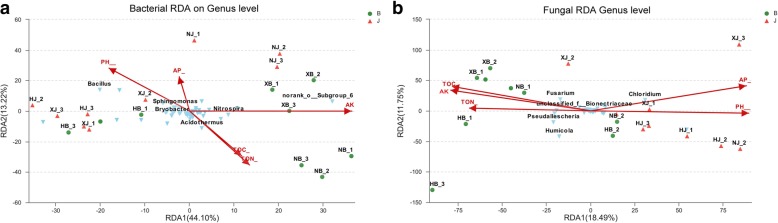
Analysis of the correlation between microorganisms and environmental variables in both types of soil samples at the level of genus. Mircoorganisms at the level of genus. **a** bacteria; **b** fungi. Environmental variables: pH,AP,AK,TOC,TON

Redundancy analysis (RDA) was employed to evaluate the soil environmental variables and phylum abundance. Our data showed that the first and second RDA components explained 30.24% of the total fungal phylum variation (Fig. 7a) and 57.32% of the total bacterial phylum variation (Fig. 7b). The diseased and disease-free soil samples were obviously differentiated by the results of fungal and bacterial RDA1 (Fig. 7a and b). For the distribution of microbial species, *Fusarium* showed negative correlations with pH and AP but positive correlations with TOC and TON. *Bacillus* had positive correlations with pH and AP but negative correlations with TOC and TON.

## Discussion

Understanding of microbial species and distributions is essential for controlling plant diseases [23]. In our study, lower colonization of *Foc* was detected in the disease-free soils, suggesting that the severity of banana *Fusarium* wilt accompanied by the increase of *Foc* colonization in the field. Previous reports have indicated that 1000 CFU/g can be considered a critical index of *Foc* diagnosis in the pot experiments [23, 24]. However, we revealed that lower than 1300 CFU/g of *Foc* colonization will not cause *Fusarium* wilt of banana in the field. It was supported by that over 1700 CFU/g of *Foc* colonization were detected in the diseased soils. This difference may be due to the complex field environment.

Although the ACE index was much higher in the diseased soils than in the disease-free soils, the species diversity based on the analysis of Shannon and Simpson had no significant differences, indicating that the species structure was similar in both types of soil samples. Higher fungal OTUs in the diseased soils may be due to an increase of *Foc* colonization, which might provoke the changes of microbial species and distributions. Thus far, a few reports have demonstrated the relationship between the incidence of fungal disease and the type of dominant genera. In our study, the dominant fungal phyla were also similar in the diseased and disease-free soils. *Ascomycota* and *Zygomycota* were the most abundant fungal phyla, accounting for 93.46 and 88.59% of total fungal ITS sequences. The abundance of *Ascomycota* in the diseased soils was much higher (*p* = 0.002) than that in the disease-free soils. It was consistent with previous study that the decrease of *Proteobacteria* and *Ascomycota* were associated with some fungal diseases suppression [25–27].

By contrast, *Mortierella*, *Fusarium*, *Pseudallescheria*, *Nectriaceae*, *Chloridium, Chaetomium*, *Humicola*, *Trichoderma* and *Ascomycota* were the dominant genera in both types of soil samples. Although the soil conditions or plant varieties could cause different distributions of the dominant fungal genera, *Fusarium* is frequently considered as one of the most dominant genera in some studies [6, 18, 28]. Nonetheless, the dominant genera *Trichoderma* and *Mortierella* were enriched in the disease-free soils, whereas *Fusarium*, *Pseudallescheria*, *Nectriacea*, *Chaetomium*, *Humicola, Sordarionmyetes* and *Gibellulopsis* demonstrated a decreasing tendency. *Trichoderma* has been widely used as a biological control agent against various pathogens [29], and although *Mortierella* has not been as a biological control agent, some antifungal and antibacterial metabolites proved to be produced by isolates of *Mortierella* [30–33]*.* Considering the only 8.30% of *Fusarium* abundance in the disease-free soils, we speculated that *Trichoderma* and *Mortierella* may be associated with suppression of banana *Fusarium* wilt disease. However, whether the regulation mechanism is through direct antagonism or resource competition, especially in the case of *Mortierella* is currently unknown.

Our results indicated that the higher abundance and diversity of bacteria were detected in the diseased soils. However, opposite results showed that a large number of OTUs were observed in the *Fusarium* wilt disease-free soils [25]. No significant difference of bacterial community distribution and diversity was exhibited in the rhizosphere soil samples of diseased and disease-free apple tree [34]. It may be caused by the field sampling time or soil microorganism changes in the presence of pathogens. Although many studies has shown that increased microbial diversity played an important role in the control of diseases [35–37], the relative abundance of several bacterial taxa is a more important indicator of disease suppression than the exclusive presence of specific bacterial taxa [17, 38]. It was supported by that most of abundant phyla such as *Proteobacteria*, *Acidobacteria*, *Chloroflexi*, *Firmicutes*, *Actinobacteria*, *Gemmatimonadetes*, *Bacteroidetes*, *Nitrospirae*, *Verrucomicrobia* and *Planctomycete* were abundant in both types of soil samples. *Proteobacteria* and *Firmicutes* were the most dominant phyla, which were similar to the previous studies on diseases infected by *Rhizoctonia solani* [38, 39]. It might be related to the different growth rate of bacteria [40, 41]. In addition, the higher abundance of *Firmicutes* in the disease-free soils supported that the disease incidence was negatively correlated with the richness of *Firmicutes* [18]. Similarly, the abundances of *Acidobacteria* and *Firmicutes* in a wheat rhizosphere were positively correlated with disease suppression [42]. Moreover, the abundances of *Bacillus*, *Lactococcus* and *Pseudomonas* in the disease-free soils were two folds than those in the diseased soils (Table 3). *Bacillus* and *Pseudomonas* were proved to be responsible for natural suppression of *Fusarium* wilt disease [25, 43–47]. *Streptococcus* was also enriched in disease-free soils probably because of the antagonist relationship between *Streptococcus* and *Fusarium oxysporum* [48], suggesting that *Streptococcus* may participate resistance to *Fusarium* wilt disease. Actually, the complex phenomenon of disease suppression in soils cannot be simply ascribed to a single bacterial taxon or group, but was governed by microbial consortia [38]. Plants and microbiota established a microenvironment on the roots with a complex interaction. A model of seven-species community evidenced that beneficial microbes inhibited the infection of phytopathogenic fungus *Fusarium verticillioides* in plant roots [11]. This model system research provides a useful method for future research on the banana-microbe interaction. However, we should fully recognize the effect of beneficial species on *Fusarium* suppression before assembling model community on banana. Comparisons of bacterial communities from banana *Fusarium* wilt diseased and diseased-free soils will prove to be essential for constructing disease suppressive soil in the future.

Based on the results of RDA analysis, disease status may accelerate soil environmental variables that affect the distribution profiles of bacterial and fungal communities [25, 49–51]. Higher AP and pH were detected in the disease-free soil samples. In addition, AP or pH exhibited a negative correlation with abundance of *Fusarium*, but was a positive correlation with abundance of *Bacillus.* A previous study reported that a high pH enhanced the suppression of *Fusarium* wilt disease [52]. Similarly, high soil AP was also associated with the inhibition incidence of stem rot disease caused by *Rhizoctonia solani* [53]. Therefore, the soil management strategies were related to the inhibition of *Fusarium* wilt disease of banana [54]. Our findings provided an evidence that higher pH and AP may enhance the inhibition of banana *Fusarium* wilt disease. It may be related to directly increased populations of beneficial genera or indirectly to altered soil nutrient availability to host plants. Additionally, TOC and TON showed significant differences between disease and diseased-free soils, with a positive correlation with the fungal taxon *Fusarium* and a negative correlation with the bacterial taxon *Bacillus*. Previous study demonstrated that bacterial and fungal communities were mainly driven by soil organic matter [55]. Indeed, due to the resource use preference of different microbes, contents of TOC and TON may alter the microbial community. For example, the soil microorganisms responded differently to the inputs of inorganic and organic fertilizers in paddy and banana soils [56–58]. Based on these findings, we can effectively suppress *Fusarium* wilt disease in practice by optimizing the structure of fertilization.

## Conclusions

We compared the distribution of the microbial communities in *Fusarium* wilt-diseased and disease-free soils. The bacterial and fungal distributions in the diseased soils were different from those in the disease-free soils. Regardles, a higher abundance of *Fusarium* was observed in the diseased soils, whereas the *Firmicutes* phylum and the *Bacillus, Lactococcus* and *Pseudomonas* genera were enriched in the disease-free soils. Regarding the fungal distribution, the abundance of *Ascomycota* in the diseased soils was much greater than that in the diseased-free soils. *Mortierella* was most dominant genera in the disease-free soils. We also found that *Bacillus* abundance, AP concentration and pH value were positively correlated with suppression of banana *Fusarium* wilt disease. These results will provide a theoretical basis for evaluating soil disease-free conditions and controlling plant diseases.

## Methods

### Preparation of soil samples

Three soil samples were selected from three banana farms designated as Nanbao (19°47′1″N and 109°51′17″E), Xinying (19°40′51″N and 109°35′58″E) and Huangtong (19°49′58″N and 109°50′58″E) in Hainan, China, respectively. The soil type is laterite soil. Banana has been planted on more than 30 hectares (ha) at each farm for 6 years. Each farm was separated by *Fusarium* wilt-diseased and disease-suppressive areas (i.e., a disease-free area). Similar field management included the banana cultivar (*Musa acuminate* AAA *Cavendish* cv. Brazil), planting density (2400 seedlings per ha), fertilization and irrigation.

Disease-free and diseased rhizosphere soil samples were collected from the three farms with the oral permission of the farm owners. Briefly, 10 individual banana trees with at least 5 m spacing were randomly selected. Each soil sample was collected from four sites of a depth of 20 cm around each tree rhizosphere using a 25 mm soil auger. Finally, a total of 40 selected soil samples from ten trees were mixed as a composite soil sample. We named the disease-free and diseased soil samples as NJ and NB from the Nanbao farm, XJ and XB from the Xinying farm, HJ and HB from the Huangtong farm, respectively. After each soil sample was ground and sieved through a 2-mm sieves, it was divided into two portions: one sample was air-dried for chemical property analysis, and the other was stored at − 70 °C for DNA extraction.

### Microbial community analysis

#### DNA extraction and PCR amplification

Microbial DNA was extracted using an E.Z.N.A.® soil DNA Kit (Omega Bio-tek, Norcross, GA, U.S.) according to the standard manufacturer’s protocol. The final DNA concentration was detected using a NanoDrop 2000 UV-vis spectrophotometer (Thermo Scientific, Wilmington, USA). The integrity of the DNA was assessed by 1% agarose gel electrophoresis.

The V3-V4 hypervariable regions of the bacterial 16S *rRNA* gene were amplified with a pair of primers 338-F (5′-ACTCCTACGGGAGGCAGCAG-3′) and 806-R (5′-GGACTACHVGGGTWTCTAAT-3′). For amplification of the fungal ITS sequences, the forward primer ITS1-F (5′-CTTGGTCATTTAGAGGAAGTAA-3′) and the reverse primer ITS1-R (5′-GGACTACHVGGGTWTCTAAT-3′) were used. The 20-μL PCR reaction systems contained 2 μL of 10× Fast*Pfu* Buffer, 2 μL of 2.5 mM dNTPs, 0.8 μL of each primer (5 μM), 0.2 μL of Fast*Pfu* Polymerase, 0.2 μL of BSA and 10 ng of template DNA. The PCR reaction was performed using a thermocycler PCR system (Thermal conditions) as follows: 3 min of denaturation at 95 °C, 27 cycles at 95 °C for 30s, 55 °C for 30s and 72 °C for 45 s, and a final elongation at 72 °C for 10 min. For the fungal ITS sequences, the same parameters were applied for 35 cycles.

After 2% agarose gel electrophoresis, the PCR products were eluted using an AxyPrep DNA Gel Extraction Kit (Axygen Biosciences, CA, USA). The DNA fragments were quantified by QuantiFluor™-ST (Promega, WI, USA) according to the manufacturer’s protocol [59].

#### Illumina MiSeq sequencing

Purified DNA fragments were pooled in equimolar amounts and paired-end sequenced (2 × 300) using the Illumina MiSeq platform (Illumina, San Diego, USA) according to standard protocols by Majorbio Bio-Pharm Technology Co. Ltd. (Shanghai, China). Raw data for bacterial 16S *rRNAs* and fungal ITS sequences were submitted to the NCBI Sequence Read Archive (SRA) database under accession number SRP132524.

#### Sequence processing

Raw sequence files were demultiplexed, quality-filtered by Trimmomatic and merged by FLASH with the following criteria: (i) these reads were truncated at any site with an average quality score < 20 over a 50-bp sliding window; (ii) primers were exactly matched allowing a 2-nucleotide mismatch, and reads containing ambiguous bases were removed; (iii) sequences with an overlap longer than 10 bp were merged according to their overlapping sequences [60]. After quality filtering and chimera removal, rarefaction curves were plotted to determine the abundance of the communities and sequencing data for each sample [59, 61]. The abundance-based coverage estimator (ACE) index, Chao richness estimator (Chao1), Shannon diversity (H) and Simpson diversity (1/D) indices were calculated using the MOTHUR package (version 1.22.2 http://www.mothur.org) with Operational Taxonomic Units (OTUs) at 0.97 level [60, 61]. OTUs were clustered with a 97% similarity cutoff using UPARSE (version 7.1 http://drive5.com/uparse/), and chimeric sequences were identified and removed using the UCHIME software [61, 62]. The classification of each 16S *rRNA* sequence and fungal ITS sequence was analyzed by the Ribosomal Database Project (RDP) Classifier algorithm (version 2.2 http://sourceforge.net/projects/rdp-classifier/) [61] against the Silva (Release128 http://www.arb-silva.de) 16S *rRNA* database and the Unite (Release 6.0 http://unite.ut.ee/index.php) database using a confidence threshold of 70% [63, 64]. To examine relationships among samples, environmental variables and frequencies of genera, RDA was carried out using CANOCO for Windows [59–61].

#### Determination of soil chemical properties

The pH of a soil water suspension (1:2.5, w/v) was measured using a glass electrode meter after shaking for 30 min. TOC and TON were determined by a dry combustion method using an Element Analyzer Vario EL (Element, Hanau, Germany). The available AP in the soil was extracted with sodium bicarbonate and then determined using a molybdenum blue method [59]. The AK in the soil was extracted with ammonium acetate and determined by a flame photometry [42, 65].

#### Pathogen quantification

A soil solution obtained from a root wash was used for pathogen quantification for all samples collected in 2015. The number of *Foc* with colony forming units (CFU) colonizing the banana rhizosphere was quantified by plating a serial dilution of rhizosphere soil suspensions onto Petri plates containing the modified Komada’s selective medium. The basal medium, including 1 g of K_2_HPO_4_, 0.5 g of MgSO_4·_7H_2_O, 0.5 g of KCl, 0.01 g of Fe-Na-EDTA, 10 g of D-Galactose (in contrast to 20 g in Komada’s original medium), 2 g of L-asparagine and 16 g of agar, was diluted in 900 mL of distilled water. The basal medium was mixed with 100 mL of a solution containing 0.9 g of PCNB (pentachloronitrobenzene, 75% WP), 0.5 g of Na_2_B_4_O_7·_10H_2_O, 0.45 g of oxgall and 0.3 g of streptomycin sulfate. The pH was adjusted to 3.8 ± 0.2 with 10% (v/v) phosphoric acid. After plating, the plates were stored at 25 °C for 10 d before colonies were counted in triplicate for each sample.

#### Statistical analysis

All parameters were analyzed using a one-way analysis of variance (ANOVA). A Tukey HSD test was performed for a multiple comparison using the SPSS Statistics 20.0 software (IBM, NewYork, USA). The distributions of bacterial and fungal species in the two types of soil samples were compared using the method of the Wilcoxon rank-sum test in the SPSS software (IBM, New York, USA).

## Abbreviations

AK: Available potassium
AP: Available phosphorus
*Foc*: *Fusarium oxysporum* f. sp. *cubense*
ITS: Internal transcribed spacer
OTU: Operational Taxonomic Units
RDA: Redundancy analysis
TOC: Total organic carbon
TON: Total organic nitrogen
